# Bias-polarity-dependent resistance switching in W/SiO_2_/Pt and W/SiO_2_/Si/Pt structures

**DOI:** 10.1038/srep22216

**Published:** 2016-02-26

**Authors:** Hao Jiang, Xiang Yuan Li, Ran Chen, Xing Long Shao, Jung Ho Yoon, Xiwen Hu, Cheol Seong Hwang, Jinshi Zhao

**Affiliations:** 1 School of Electronics Information Engineering, Tianjin Key Laboratory of Film Electronic & Communication Devices, Tianjin University of Technology, Tianjin 300384, China; 2 Department of Materials Science and Engineering and Inter-university Semiconductor Research Center, Seoul National University, Seoul 151-744, Korea

## Abstract

SiO_2_ is the most significantly used insulator layer in semiconductor devices. Its functionality was recently extended to resistance switching random access memory, where the defective SiO_2_ played an active role as the resistance switching (RS) layer. In this report, the bias-polarity-dependent RS behaviours in the top electrode W-sputtered SiO_2_-bottom electrode Pt (W/SiO_2_/Pt) structure were examined based on the current-voltage (I-V) sweep. When the memory cell was electroformed with a negative bias applied to the W electrode, the memory cell showed a typical electronic switching mechanism with a resistance ratio of ~100 and high reliability. For electroforming with opposite bias polarity, typical ionic-defect-mediated (conducting filament) RS was observed with lower reliability. Such distinctive RS mechanisms depending on the electroforming-bias polarity could be further confirmed using the light illumination study. Devices with similar electrode structures with a thin intervening Si layer between the SiO_2_ and Pt electrode, to improve the RS film morphology (root-mean-squared roughness of ~1.7 nm), were also fabricated. Their RS performances were almost identical to that of the single-layer SiO_2_ sample with very high roughness (root-mean-squared roughness of ~10 nm), suggesting that the reported RS behaviours were inherent to the material property.

In semiconductor devices, SiO_2_ has very diverse configurations in terms of its thickness, resistivity, density, and dielectric constant depending on its purpose of use. Despite the diverse aspects of SiO_2_, it has been regarded as a completely passive component of semiconductor devices due to its role as an insulator or a dielectric layer in capacitive components. However, as the resistance-switching random access memory (RRAM) is becoming one of the leading contenders for the next-generation non-volatile memory, SiO_2_ along with other highly diverse metal oxides were tested as active resistance-switching (RS) layers. Several transition metal oxides (TMOs), such as TiO_2_, NiO, Ta_2_O_5_, and HfO_2_, have been tested as RS layers because the multi-valence nature of the transition metals would render the RS in these materials easily achieved^1^,^2^,^3^,^4^. Additionally, the so-called voltage-time dilemma from typical RS materials triggered interest in finding alternative RS materials, and several groups had tested SiO_2_^5^,^6^,^7^. Due to the mostly ionic bonding in the TMO RS materials, electric-field-driven ion migration is easier when the material contains an appropriate density of ionic defects. On the other hand, SiO_2_ is more covalently bonded and lacks long-range order. These differences in properties provide an SiO_2_ memory with several merits over other TMO RS materials, as discussed shortly.

The commonly accepted RS mechanism in many TMOs is the formation and rupture of conducting filaments (CFs), which are either an aggregation of defects, such as oxygen vacancy (V_O_), the nano-scale conducting phase (e.g., the Magnéli phase in TiO_2_), or the metallic filament (e.g., Cu) in the electrochemical metallization (ECM) cell^8^,^9^,^10^. No matter what the detailed nature of these CFs is, the involvement of ionic defects, i.e., electric-field-induced defect generation and migration (assisted by Joule heating) as well as thermal motion (for rupturing in non-polar RS), is the critical factor of memory operation. For the case of SiO_2_, the pioneering work by Tour’s group showed that the RS mechanism involved the formation of Si clusters during the electroforming and subsequent phase transition between the metallic and semiconducting phases depending on the bias sequence^11^. This is a critical feature of the SiO_2_-based RS system, which differentiates it from other valance-change-based or thermochemical-reaction-based RS mechanisms of many TMO materials, where the reversible redox reaction of the constituent metal ions is responsible for the RS mechanism. The characteristic feature of the non-polar switching of the SiO_2_-based RS material, where the voltage for the reset [the switching from a low-resistance state (LRS) to a high-resistance state (HRS), or V_reset_] is higher than the voltage for the set (the switching from an HRS to an LRS, or V_set_) may be ascribed to such phase-transition-related switching mechanism, whereas V_set_ is higher than V_reset_ in usual TMO-based RS systems^11^. The reason why such behaviour, i.e., a higher V_reset_ than V_set_, is peculiar is as follows. In general, almost all RRAM cells have a series resistance component due to, for example, contact resistance and interconnection wire resistance, which take up a certain portion of the applied voltage (V_a_) during the RS operation. For the reset, the RRAM cell is initially in an LRS, so it takes a small portion of V_a_ before switching, and a substantial portion of V_a_ is applied to the series resistance. However, after the memory cell has been reset, its resistance becomes much higher. Then the voltage over the memory increases abruptly because the portion of the voltage applied to the series resistor is now dumped into the memory. If the magnitude of the series resistor was high, the dumped voltage was also high, which could make the memory cell voltage now even higher than V_set_. This instantaneously sets the memory cell again, which means stable resetting is not probable in this case. Kim *et al.* elucidated the details of such difficulty^12^. Therefore, if the V_reset_ of a certain memory cell is higher than its V_set_, such problem could be further aggravated. However, it is instructive to recall that phase change memory materials generally have a higher V_reset_ than V_set_, where the thermal-energy-induced reversible phase transitions of the phase change materials are responsible for the repeatable switching^13^. As the RS in the SiO_2_-based RRAM is ascribed to the reversible phase transition of the Si clusters between the metallic and semiconducting phases, there might be a chance that a similar mechanism works, although it cannot be precisely understood yet. The reactively sputtered SiO_2_ film used in this study also showed a characteristic feature similar to that in the case of the non-polar switching operation. However, its reliability was quite low (only several tens of switching cycles were possible), and thus, it was not seriously studied. Rather, the bipolar-type operation was attempted, which showed at least ~3,000 times reliable switching even in the worst case.

These ionic-defect-mediated RS’s generally had reliability concerns and uniformity issues, although several great improvements were also achieved during the past decade^14^,^15^,^16^,^17^,^18^,^19^. On the other hand, the electronic bipolar resistance switching (eBRS) mechanism has emerged more recently, where trap-mediated carrier trapping and detrapping were suggested to be the primary RS mechanism^20^,^21^,^22^. Such eBRS mechanism operates mainly when an asymmetric potential barrier is present, which facilitates carrier trapping under one bias polarity, and carrier detrapping under the opposite bias polarity. Kim *et al.* explained such mechanism in great detail for the case of the TiO_2_ memory cell^21^. Due to this characteristic trapping/detrapping mechanism of such RS behaviour, it must be naturally bipolar, which means it is mainly a field-driven phenomenon with minimal involvement of thermal energy. Therefore, eBRS can be a viable way to achieve the goals of low power consumption, high-speed operation, and high reliability of RRAM^23^. However, according to earlier reports on eBRS in the TMO system, the above-mentioned advantages are not always valid because the traps related to the ionic defects are unstable during repeated RS, making the time and switching cycle dependent drift of the switching parameters, such as the resistance ratio and the switching voltages^24^,^25^,^26^. In this regard, defective SiO_2_ could be a possible option for such eBRS because its defect configuration may be influenced less by the external electrical stress due to its higher bonding energy and lower ionic character, compared with the other TMOs. Chen’s group^27^ reported remarkable work in this field, where dispersed Pt nano-clusters provided carrier trapping centers in SiO_2_.

In this study, the authors attempted to explore the active role of the reactively sputtered SiO_2_ layer as a charge trapping layer in eBRS and as a medium for CF formation in ionic BRS (iBRS). For this purpose, a certain asymmetry was necessary regarding carrier migration, which was provided by adopting an asymmetric electrode configuration: a low-work-function W-top electrode (TE) and a high-work-function Pt-bottom electrode (BE), which had a very low chance of being incorporated into the SiO_2_ so that the possibility of the ECM-type RS can be ignored. The reactive sputtering process generated defects with a sufficiently high density in the dielectric layer to make the RS quite fluent even without doping of any other metallic elements. The highly stable amorphousness of SiO_2_ eliminated the concern of (local) crystallization of the material during repeated switching under severe electric test conditions. By changing the bias polarity of the first electroforming step, eBRS and iBRS were acquired, and their characteristics were compared. To further elucidate whether the observed electroforming-bias-dependent switching behaviour is due to the material-inherent properties or film morphology-dependent extrinsic factors, films with similar chemical properties but with distinctive morphologies were prepared by inserting a thin (2 nm) Si layer between the sputtered SiO_2_ and Pt bottom layers. It was concluded that the observed RS performances have little relevance to the film morphologies, which could be a critical ingredient of integrated RRAM device fabrication.

## Results and Discussions

**Figure 1:**
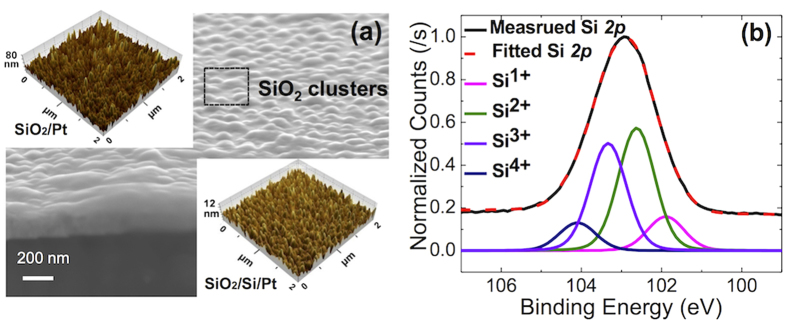
(**a**) The bird’s eye view SEM image of the sputtered SiO_2_ on Pt film. The upper and the lower inset figures are the AFM image of SiO_2_/Pt and SiO_2_/Si/Pt, respectively. (**b**) The XPS results of Si2*p* core-level spectra of SiO_2_/Pt.

Figure 1a shows a bird’s eye view scanning electron microscopy (SEM) image of the ~15 nm-thick sputtered SiO_2_ film deposited directly on the Pt electrode. The film had a rough surface morphology perhaps due to the non-wetting property of SiO_2_ on Pt. The atomic force microscopy (AFM) image in the upper inset corroborates the rough SEM image and the root-mean-squared (RMS) roughness of the film as ~10 nm (from a 2 × 2μm^2^ area), versus that of the Pt substrate of ~1 nm. By contrast, the SiO_2_ film with the identical thickness grown on the 2 nm-thick Si/Pt electrode showed a much lower RMS roughness of ~1.8 nm (in the lower-inset AFM image), which suggests that the intervening thin Si layer greatly improved the wetting property and thus, the morphology of the growing SiO_2_ film. Although the surface morphology of the Si/Pt substrate was not checked via AFM, the smooth morphology of the SiO_2_ film grown on top suggested that the Si/Pt had a smooth and uniform morphology. Figure 1b shows the X-ray photoelectron spectroscopy (XPS) Si2*p* core-level spectra of the film on the bare Pt electrode. The XPS showed that the film had a substantial amount of Si-suboxides (Si^+^, Si^2+^, and Si^3+^), in addition to regular SiO_2_ (Si^4+^). The O/Si ratio estimated from the O1s and Si 2*p* peak areas was not too different from that of the thermal SiO_2_. The SiO_2_ film on the Si/Pt electrode showed almost identical XPS spectra (data not shown), which is reasonable considering the very low diffusion rate of Si in SiO_2_ at the processing temperature. It could also be possible that the very thin Si was (partly) oxidized during the subsequent SiO_2_ deposition step. Therefore, comparing the RS performance of these two films could be a feasible way to confirm the influence of the film morphology on the RS mechanism.

**Figure 2:**
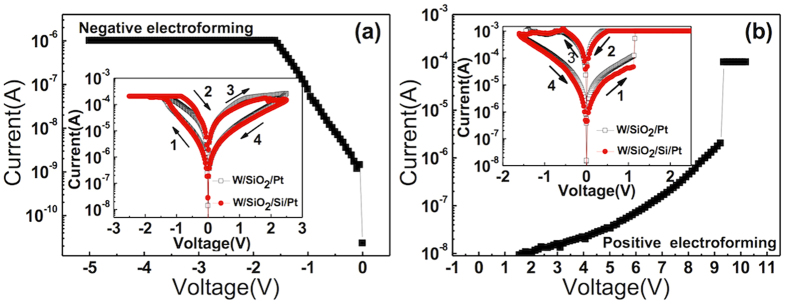
The electroforming I-V curve of W/SiO_2_/Pt samples under (a) negative bias and (b) positive biases. The subsequent BRS I-V sweep curves of W/SiO_2_/Pt and W/SiO_2_/Si/Pt are included as the insets in Fig. 2(a,b), respectively.

The W/SiO_2_/Pt sample showed highly asymmetric current-voltage (I-V) curves in the pristine state, which can be expected from the asymmetric configuration of the electrode (W: work function of 4.32–5.22 eV, and Pt: work function of 5.12–5.93 eV). Figure 2a,b show the electroforming curves (the I-V curves in the pristine state) under negative and positive biases, respectively. Under the negative bias, the current started to increase gradually at ~ −0.1 V, which reflected a low Schottky barrier height (SBH) at the W/SiO_2_ interface; whereas under the positive bias, the current first remained very low up to ~9 V, then suddenly increased, revealing an abrupt electroforming that could be ascribed to a higher SBH at the Pt/SiO_2_ interface than at the W/SiO_2_ interface. The W/SiO_2_/Si/Pt sample also showed an asymmetric I-V curve in the pristine state and distinctive electroforming behaviour, which were very similar to those of the W/SiO_2_/Pt sample. Therefore, it was expected that the subsequent RS I-V curves at both electroforming-bias polarities would be similar, which was indeed the case, as shown below. After several trials with different current compliance (I_cc_) values, the suitable I_cc_ values were settled at 1 μA and 100 μA for the negative and positive biases, respectively. It exhibits hard breakdown if I_cc_ was set above the transition point for negative electroforming case. The subsequent BRS I-V sweep curves are included in Fig. 2 as the inset figures, which show overlapping of the I-V sweep results from the W/SiO_2_/Pt (black square symbol) and W/SiO_2_/Si/Pt (red circle symbol) samples. It can be immediately understood that in both cases, the set and reset could be achieved under the negative (positive) and positive (negative) bias polarities in Fig. 2a,b, respectively, which coincided with the electroforming direction. Another interesting finding is that the I_cc_ level for electroforming was much lower than the I_cc_ for the set and maximum reset currents during the subsequent I-V sweep for both the negatively and positively electroformed cases, whereas they are usually similar for general TMO RRAMs^28^,^29^. In fact, the memory cell remained at HRS even after the electroforming, unlike with other TMOs^28^,^29^,^30^,^31^. This finding suggests that the role of electroforming in those cases could be different from that of other RRAM cells that use TMO. It is believed that the role of negative electroforming in the W/SiO_2_/Pt structure was to non-uniformly lower the SBH at the W/SiO_2_, which will be demonstrated detail through are-dependency experimental data, and increased the electron injection from W to the SiO_2_ layer. Although the extreme asymmetry in the I-V shape with respect to the bias polarity of the pristine state became much lower after the negative electroforming, there was still remaining asymmetry in I-V of the LRS (inset figure of Fig. 2a). The general current level of LRS was, of course, much higher than the pristine state. These findings suggested that, in this case, the Fermi level of the SiO_2_ layer became higher after the injected carriers were trapped inside the oxide layer, making SBH at both interfaces lower. Even under this circumstance, the SBH at the Pt/SiO_2_ interface was still slightly higher than that at the W/SiO_2_ interface, resulting in the I-V asymmetry even at LRS. This is a reasonable consequence of the higher work function of Pt than that of W, and the I-V asymmetry is the critical factor necessary to induce the eBRS.

By contrast, the positive electroforming induced almost complete local breakdown of SBH at both the Pt/SiO_2_ and W/SiO_2_ interfaces by the formation of CFs, and that the subsequent I-V sweep induced additional changes in the film, as discussed below.

**Figure 3:**
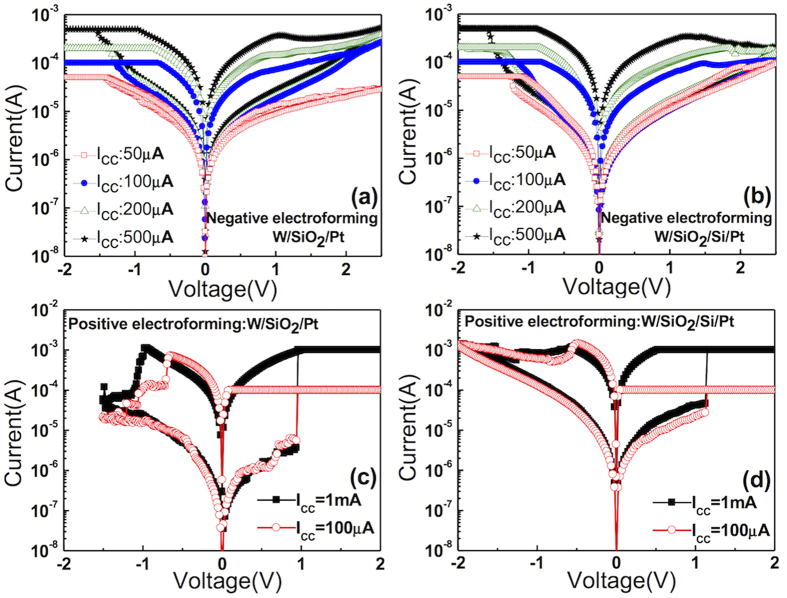
The RS I-V curves with various I_cc_ (50 μA −500 μA) after negative electroforming in (a) W/SiO_2_/Pt and (b) W/SiO_2_/Si/Pt. The I-V curves after positive electroforming with I_cc_ of 100 μA and 1 mA in (c) W/SiO_2_/Pt and (d) W/SiO_2_/Si/Pt.

Figure 3a shows the evolution of I-V after the first electroforming with the increasing I_cc_ (from 50 μA to 500 μA) of the W/SiO_2_/Pt sample, wherein the I-V sweep always started by sweeping into a negative bias. When the I_cc_ was lower than ~100 μA, there was only a small resistance ratio between the LRS and the HRS in both bias polarities. These features of RS and the relatively smooth switching I-V curve shape with a small resistance ratio for the negatively electroformed cell suggested that the RS mechanism can be ascribed to the electron trapping and detrapping (eBRS). After the electroforming, when the W TE was negatively biased, electrons were fluently injected into SiO_2_ and were trapped via the location where SBH was broken locally. Then the sample state changed from HRS to LRS. Under a positive bias application, the trapped electrons were detrapped, whereas the higher SBH at the Pt/SiO_2_ interface suppressed the electron injection from Pt BE. Figure 3b shows the evolution of I-V after the first electroforming with the increasing I_cc_ (from 50 μA to 500 μA) of the W/SiO_2_/Si/Pt sample, which showed behaviours almost identical to those shown in Fig. 3a. Figure 3c shows the I-V curves of the positively electroformed W/SiO_2_/Pt cell with I_cc_ values of 100 μA and 1 mA during the set operation after the electroforming. The I-V shapes are very similar to the general BRS curve shape of other TMO-based RRAM cells, which suggests the possible involvement of CFs in such case^32^,^33^,^34^. The CFs were most likely composed of Si clusters or a V_O_-clustered semiconducting phase^11^,^27^. Figure 3d shows the I-V curves of the positively electroformed W/SiO_2_/Si/Pt cell with an I_cc_ identical to that in Fig. 3c. Despite their slight discrepancy, especially in the abruptness of the reset in the negative bias region, they showed RS features basically similar to those shown in Fig. 3c. The results shown in Fig. 3 suggest that the thin intervening Si layer at the Pt bottom electrode interface only marginally influenced the RS characteristics of the memory cell.

**Figure 4:**
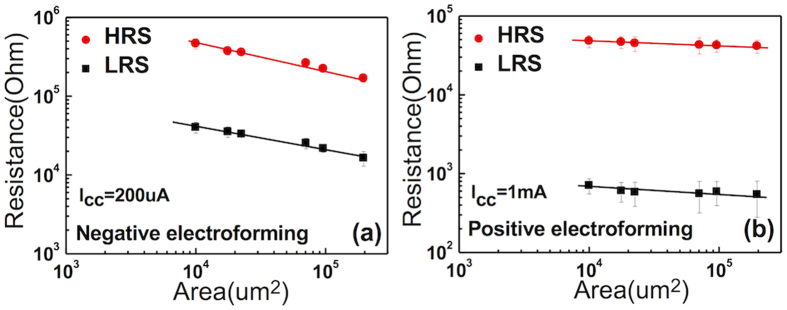
The electrode area-dependent resistance of LRS and HRS for (a) the negative electroforming case, and (b) the positive electroforming case.

Figure 4a,b show the electrode area-dependent (W TE) resistance of the W/SiO_2_/Si/Pt sample for the negative (I_cc_ of 200 μA) and positive (I_cc_ of 1 mA) electroforming cases, respectively. The memory states with negative electroforming showed a certain area-dependency for both LRS and HRS, which suggests that the switching occurred across the entire area, but was not completely uniform, i.e. the electrode area-dependency of the switching behaviour was in between the completely uniform and completely localized switching behaviours. This is consistent with the idea that negative electroforming locally destroys the Schottky barrier at the W/SiO_2_ interface, and that the injected carriers may be diffusely trapped at the trap centers which located rather uniformly across the entire electrode area. In contrast, the positively electroformed memory cell showed almost no electrode-area dependency for both LRS and HRS, which is consistent with the highly localized CF-formation and rupture phenomenon in many other TMO-based RRAMs. It was also noted that the negatively electroformed cases had a much lower data scatter than the positively electroformed case. This is consistent with the general idea of the random nature of the CF-mediated ionic BRS (iBRS) mechanism (in the positively electroformed case), unlike the slightly more area-type eBRS^35^.

**Figure 5:**
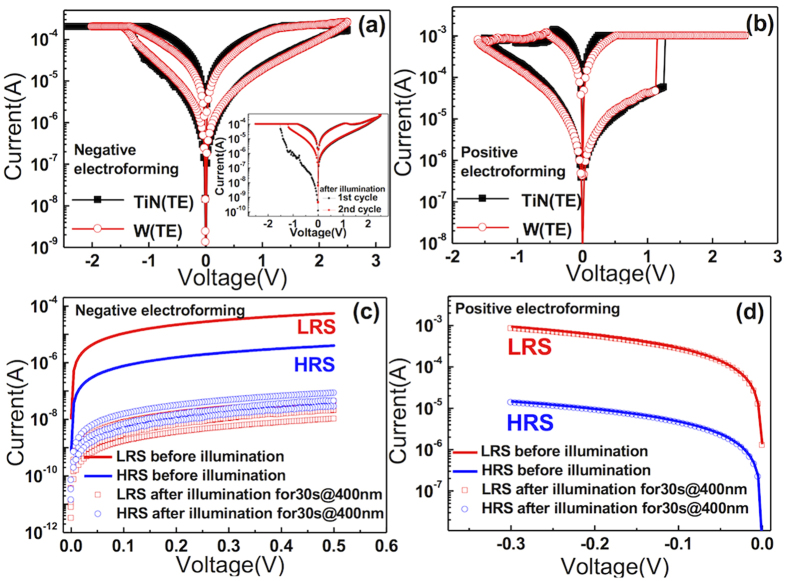
(**a,b**) show the RS I-V curves with either W (red symbol) or TiN (black symbol) TE after negative electroforming and positive electroforming, respectively. The inset of (**a**) is RS I-V curves after the light illumination - the black curve corresponded to the I-V curve immediately after the illumination. I-V curves of LRS and HRS before illumination and after illumination are shown in (**c**) the negative electroforming case and (**d**) the positive electroforming case.

The eBRS and iBRS mechanisms for the negatively and positively electroformed cases were further confirmed by the photon-induced detrapping experiment. In eBRS, the set switching is generally ascribed to the filling of deep traps with injected carriers during the set process. When the deep traps are filled with carriers (supposed to be electrons), the filled traps cannot interfere with further injected carriers, so the sample shows LRS during the subsequent reading step. In contrast, the reverse bias application during the reset process detraps the trapped carriers, whereas the high SBH at the Pt/SiO_2_ interface in this case suppresses the carrier injection from the opposite electrode. When the LRS sample is illuminated with high-energy photons, the carriers can be photon-excited and detrapped, which would induce the sample to return to the HRS even without the reset bias application. Therefore, such photon-induced resistance increase could be a critical evidence of the soundness of the eBRS mechanism, which has been proven in the case of TiO_2_^15^. Nevertheless, the W TE sample cannot be used for this purpose because the photon cannot penetrate the 200 nm-thick W TE. Therefore, a 5 nm-thick TiN electrode, which is thin enough to allow light penetration, was deposited, and its electrical properties were checked. Figure 5a shows the RS I-V curves of the SiO_2_/Si/Pt sample with either a thick W or thin TiN electrode after the negative electroforming, which confirmed that the two structures showed similar switching characteristics. Figure 5c shows the I-V curves in the positive bias region for LRS and HRS before (solid lines) and after (open and closed square symbols) the light illumination with a wavelength of 400 nm (photon energy = 3.1 eV) for 30 sec with the TiN TE. The current decreased significantly for both LRS and HRS after the light illumination, which suggests that photon-induced detrapping occurred. It is interesting that the HRS also showed a large current decrease, which indicates that a substantial portion of carriers remain trapped even after the reset bias application. This could be partly due to the incompletely suppressed carrier injection from the Pt electrode and partly due to the insufficient voltage stress for detrapping the deeply trapped carriers. The photon-excitation experiment was also performed with longer wavelengths but it did not work, which suggests that the trap depth in SiO_2_ is much deeper than in ca. TiO_2_ (<1 eV), which is consistent with the much wider band gap of SiO_2_. It was noted that the maximum reset voltage in Fig. 5a was limited to 2.5 V, whereas the trap depth was estimated to have been at least 3.1 eV (400 nm wavelength), under which circumstance the voltage-induced detrapping could not be sufficient. The increases in the reset voltage further decreased the HRS current to a certain degree but not as efficiently as did the light illumination. The inset in Fig. 5a shows the RS curves after the light illumination. The black curve corresponds to the I-V curve immediately after the illumination, which was first attempted with a negative bias. Even though the sample showed a very low current immediately after the illumination, which is almost comparable to the pristine sample, once it was switched to LRS with the application of a sufficient negative bias (−2.5 V), it recovered the previous I-V switching current level, and the subsequent I-V sweep showed current levels for both LRS and HRS almost identical to those before the light illumination. This implied that the light illumination did not alter the physical status of the traps, but only the trapped carriers were detrapped. In contrast, the positively electroformed samples showed a completely disparate response to the light illumination experiment, as shown in Fig. 5b,d.

Figure 5b shows the switching I-V curves after the positive electroforming with either W (the red symbol) or TiN (the black symbol) TE, which again confirmed that they were almost identical. Figure 5d shows the I-V curves in the negative bias region before (lines) and after (dots) the light illumination under the same condition as that shown in Fig. 5c. It can be immediately understood that the light illumination could not induce any alteration in the resistance state for both LRS and HRS. This is consistent with the assertion that the RS in the positively electroformed sample is mediated by the discrete CF-related mechanism, which has little relevance to the carrier trapping. The switching mechanisms were further confirmed by the electrical conduction mechanism analysis, as shown in the next section.

**Figure 6:**
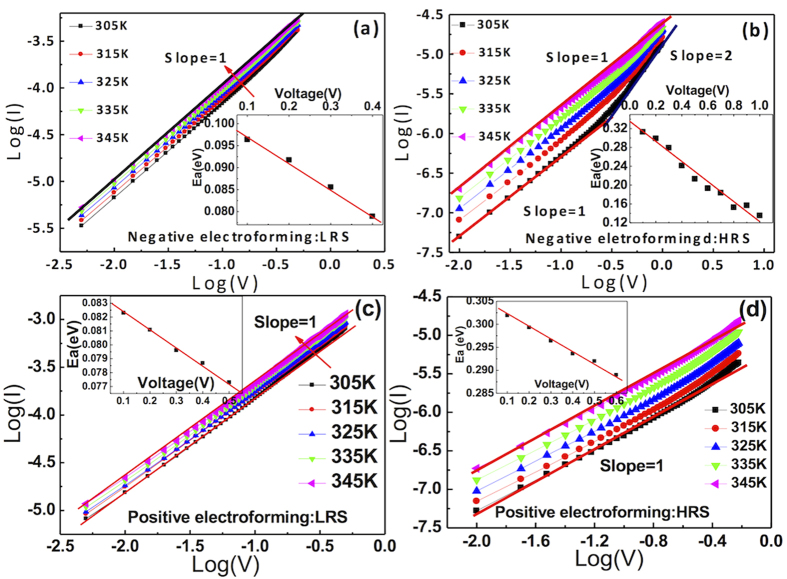
The log I – log V curve of W/SiO_2_/Pt sample measured from 305 K to 345 K at (a) LRS of negative electroformed sample, (b) HRS of negative electroformed sample, (c) LRS of positive electroformed sample, and (d) HRS of positive electroformed sample. The activation energy (E_a_) at each voltage is summarized in the inset figures.

Figure 6a shows the log I – log V curves of the W/SiO_2_/Pt sample of the LRS of the negatively electroformed sample measured at temperatures that ranged from 305 K to 345 K. The slopes of the best-linear-fitted graphs were almost close to 1, and the current level slightly increased with the increasing temperature, which suggests that the conduction occurred via the hopping mechanism. The Arrhenius-type plot showed that the activation energy was 0.08–0.095 eV at the read voltage range of 0.1–0.4 V (inset figure). This finding indicated that the trapped electrons moved along with the voltage via the hopping mechanism. Figure 6b shows the log I – log V curves of the same sample measured at the same temperature range for the HRS. At lower temperatures (305 and 315 K), the I-V showed a trend similar to that of the space charge limited current (SCLC), i.e., at a low (absolute) voltage, the slope was close to 1, but as the voltage increased (as the carrier injection increased), the slope became close to 2, due to interactions between the trapped carriers^21^,^36^,^37^,^38^. As the temperature increased, the current increased at a faster rate than did the LRS, and the ohmic current flowed over the entire tested voltage region. The estimated activation energy was 0.14–0.31 eV at the read voltage of 1.0–0.1 V (inset figure). This result suggested that the current transport at the HRS was mediated by slightly deeper traps, the values of which decreased with an increasing bias due to the Poole-Frenkel-like effect. Similar electrical conduction mechanism studies were performed for the W/SiO_2_/Si/Pt sample, and almost identical conclusions were achieved (data not shown).

For the positively electroformed memory cell with both W/SiO_2_/Pt and W/SiO_2_/Si/Pt structures, similar conduction mechanism analyses were performed for both the LRS and HRS. Figure 6c reveals that the LRS was characterized by the hopping conduction with a small activation energy of ~0.083 eV (inset figure). This meant that the CF had a hopping conduction property with very small activation energy, which might be consistent with the assertion that the CF is composed of relatively disordered Si clusters. The HRS also showed a hopping conduction mechanism (Fig. 6d) but with a much higher activation energy of ~0.28–0.30 eV (inset figure). This implies that the CF, supposedly Si clusters, in the memory cell has a high density of traps with a relatively large distribution of energy values within the band gap, whereas the traps with shallow and deep levels mediated the electrical conduction in the LRS and the HRS, respectively. Next, the electrical performance of the RS cell of the negatively and positively electroformed samples was evaluated.

**Figure 7:**
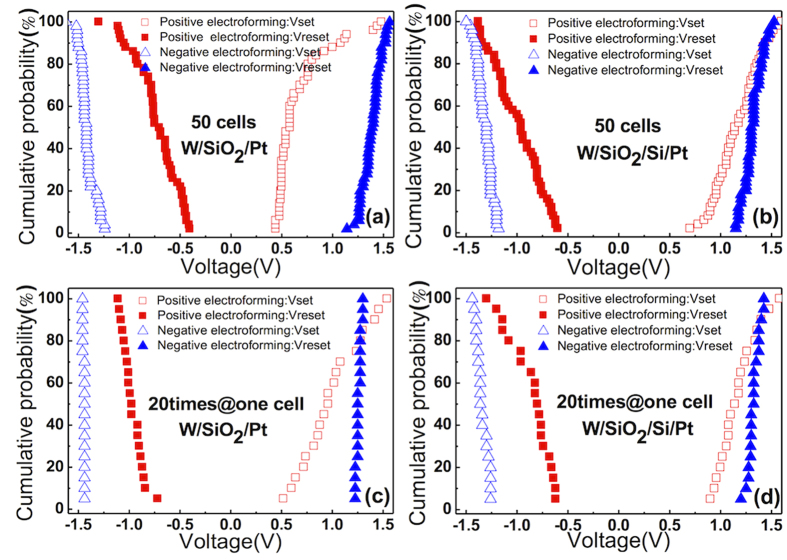
The cumulative probability graphs of V_set_ and V_reset_ with 50 cells and the repeating 20 times in one cell for the negative electroforming and the positive electroforming case, respectively, corresponding to (a,c) W/SiO_2_/Pt and (b,d) W/SiO_2_/Si/Pt.

Figure 7a,b show the V_set_ and V_reset_ uniformity of the W/SiO_2_/Pt and W/SiO_2_/Si/Pt structures, respectively, when the electroforming was performed with negative (triangle symbol) and positive (square symbol) biases, wherein 50 cells were tested for each condition. The negatively electroformed cell had a much higher uniformity with a higher set/reset voltage window than the positively electroformed cell. Figure 7c,d show the V_set_ and V_reset_ uniformity of one memory cell for each condition when they were tested 20 times, which also confirmed that the negatively electroformed samples had a higher uniformity. These results are consistent with the trends in the standard deviation shown in Fig. 4.

**Figure 8:**
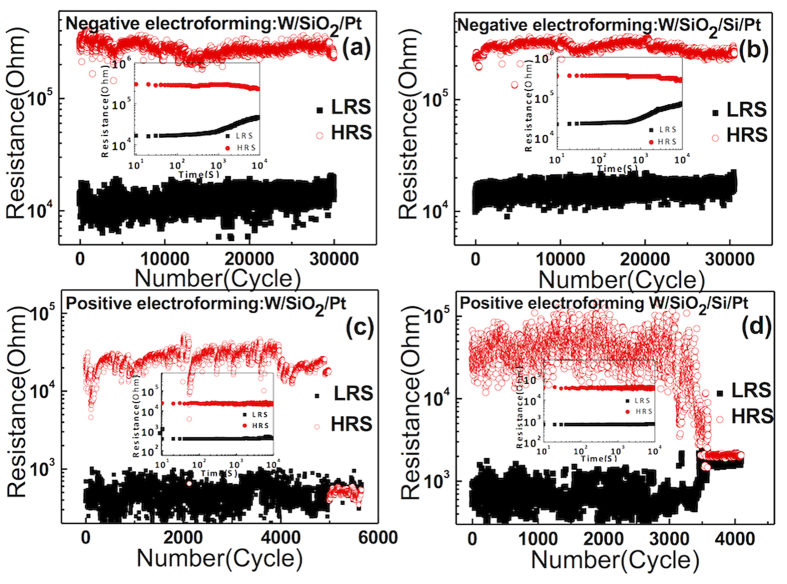
The endurance test results of the W/SiO_2_/Pt and W/SiO_2_/Si/Pt achieved from the repeated I-V sweeps for (a,b) negatively electroformed cells, and (c,d) the positively electroformed cells. The insets are corresponding retention data measured at 85 °C.

**Figure 9:**
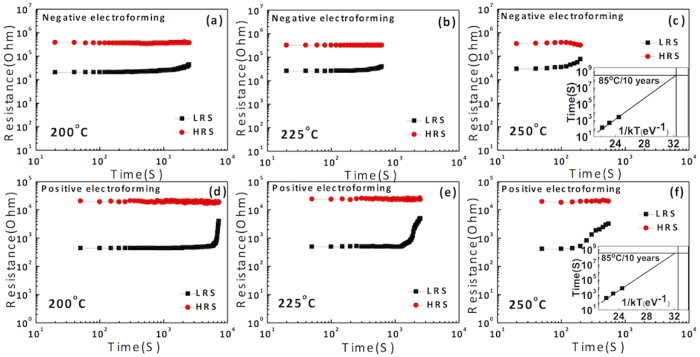
The retention data measured at different temperatures for (a–c) negative electroformed cell, and for (d–f) positively electroformed cells. The insets of (**c,f**) are the Arrhenius plots, which indicate the data for both electronic and ionic switching could be maintained for 10 years at 85 °C.

Figure 8a,b show the endurance test results of the W/SiO_2_/Pt and W/SiO_2_/Si/Pt structures, respectively, obtained via repeated I-V sweeps for the negatively electroformed cells. Here, the resistance values were estimated at 0.1 V. Both samples showed high reliability for up to 30,000 switching cycles without any sign of degradation. It is believed that the actual maximum endurance cycle number could be much higher, but it cannot be tested through such time-consuming I-V sweep tests. It has to be noted that such I-V sweep test has a much harsher condition than the pulse-switching type test, although the latter is closer to the actual device operation. In contrast, the positively electroformed cell showed no more switching after ~5,000 and 3,500 cycles for the W/SiO_2_/Pt and W/SiO_2_/Si/Pt structures, respectively, as shown in Fig. 8c,d, which suggests the much lower reliability of the CF-mediated iBRS mechanism. When the devices failed, they always stayed at the LRS without recovering the HRS, which suggests the occurrence of a hard breakdown. The retention test results at 85 ^o^C for the identical sample conditions are shown in the insets in Fig. 8. While the positively electroformed cell showed stable retention of both states, as expected (see the insets in Fig. 8c,d), the negatively electroformed cell showed certain limitations, especially for the LRS (see the insets in Fig. 8a,b), which can be understood from the carrier trapping-mediated mechanism^39^,^40^. In this case, circuit level supplementation, i.e., reading of the data after a certain period of time and its rewriting, would be necessary. Figure 9 shows the retention data for W/SiO_2_/Pt sample, measured at temperatures of 200 °C (a,d), 225 °C (b,e) and 250 °C (c,f) for (negative, and positive) electroforming cases. Here, the read intervals for negative and positive electroformed samples were 20 s and 50 s, respectively. After certain time (2400 s, 460 s, and 120 s in Fig. 9a–c, respectively), the resistance ratio became lower than 10, indicating the data was losing. For positively electroformed cell, the time when the resistance ratio becomes lower than 10 was generally longer (7000 s, 1400 s, and 350 s at 200 ^o^C, 225 ^o^C and 250 °C, respectively), suggesting higher retention. These retention behaviors were plotted according to the Arrhenius form (insets Fig. 9c,f), and extrapolated to check if the retention time at 85 ^o^C could be longer than 10 years. Both cells showed 10-year retention time where the positively electroformed cell showed the 10-year retention time even at a slightly higher temperature (90 °C).

In conclusion, the RS properties of W/SiO_2_/Pt and W/SiO_2_/Si/Pt memory cells were examined depending on the direction of the electroforming. When the electroforming was accomplished with the application of a negative bias to the W TE, the RS was achieved fluently and reliably with high uniformity via the electronic switching mechanism, which was confirmed by the carrier detrapping experiment with light illumination (400 nm wavelength). The trap depth was estimated to have been at least ~3.1 eV. In contrast to the eBRS mechanism acquired from mostly ionic materials such as TiO_2_, the more covalent nature and stronger bonding energy of SiO_2_ prohibited the variation of the trap configuration during the repeated bias application. These effects resulted in very high reliability, which has not been achieved from any other eBRS system. In contrast, in the electroforming with the application of a positive bias to the W TE, the usual ionic-defect-mediated bipolar switching mechanism was observed, in which the electrical conduction was hardly influenced by the light illumination. In contrast to the previous non-polar ionic switching, wherein the phase transition of Si nano-clusters was responsible for the switching, the bipolar nature of the iBRS in this study suggested that it is mediated by the reversible oxidation and reduction of the Si clusters mediated by the bias polarity. During the electroforming and set switching under the positive bias condition, oxygen ions were migrated toward the W electrode, which then moved back to the SiO_2_ layer during the subsequent reset in the negative bias region and reoxidized the Si-cluster CFs. This is similar to the general CF-mediated ionic bipolar switching in many other TMO RRAMs, and they thus share common features in terms of performance. The two samples with highly distinctive morphological properties, i.e., the SiO_2_ films on the Pt and Si/Pt layer, had roughness values of ~10 nm and ~1.8 nm, respectively, and showed no significant difference in their RS performance and electrical conduction behaviours, which suggest that the observed RS properties are inherent in the material properties of the grown SiO_2_ film. Overall, the reactively sputtered SiO_2_ film showed great potential as an RS material for RRAM, especially when it was negatively electroformed.

## Methods

A 120 nm-thick Pt thin film was grown on an SiO_2_/Si wafer using the DC sputtering method as a continuous BE. An approximately 10–15 nm-thick SiO_2_ layer was reactively sputtered on Pt BE using an undoped Si wafer as the target, with a sputtering power of 100 W, Ar : O_2_ ratio = 29.7 : 0.3 standard cubic centimetre per min., and a total pressure of 0.5 Pa. Under these sputtering conditions of the SiO_2_ film, the wetting property of the growing SiO_2_ layer on Pt was very low, which made the film extremely rough (RMS roughness: ~10 nm for the 15 nm-thick film). To mitigate this problem by improving the wetting property of the SiO_2_, a thin (2 nm-thick) Si layer was *in-situ* sputtered before the SiO_2_ sputtering, which drastically decreased the RMS roughness to ~1.7 nm for the similarly thick SiO_2_. Then a 200 nm-thick W layer was deposited via dc sputtering from the W metal target through a metal shadow mask (opening diameter: 0.2 mm). Electrical tests were performed at temperatures that ranged from 305 K to 345 K using an Agilent B1500 in the voltage sweep mode to achieve the switching I-V curves. During the I-V sweeps, the W TE was biased, whereas the Pt BE was grounded. To further elucidate the eBRS mechanism, which differs from iBRS, light-induced detrapping experiments were also performed, as had been done for the TiO_2_-based eBRS^15^. For this experiment, light must penetrate through the TE, which is not feasible for the 200 nm-thick W layer, so a 5 nm-thick TiN layer was reactively sputter-deposited on the SiO_2_ film through an identical shadow mask. The 5 nm-thick TiN layer was thin enough to allow the light to pass through, while still thick enough to form a stable electrical contact so as to electrically switch the films to either HRS or LRS under a positive or negative bias. While the trap in the TiO_2_ was relatively shallow (<1 eV), which is understandable from its lower band gap (~3 eV) so that the useful wavelength of the shone light could be relatively long (1200 nm), the trap in the SiO_2_ was much deeper, and only 400 nm of the available wavelength (400–700 nm) of the equipment (Hitachi F-4600 Analysis System) was useful.

The deposited SiO_2_ film was not thermally treated. Its surface morphology and chemical properties were examined via SEM (ZEISS, MERLIN Compact), AFM (JEOL, JSPM 5300), and XPS (ThermoVG, Sigma Probe), respectively. The x-ray diffraction showed that the film was amorphous.

## Additional Information

**How to cite this article**: Jiang, H. *et al.* Bias-polarity-dependent resistance switching in W/SiO_2_/Pt and W/SiO_2_/Si/Pt structures. *Sci. Rep.* **6**, 22216; doi: 10.1038/srep22216 (2016).
